# Boosting Akt Pathway by Rupatadine Modulates Th17/Tregs Balance for Attenuation of Isoproterenol-Induced Heart Failure in Rats

**DOI:** 10.3389/fphar.2021.651150

**Published:** 2021-04-30

**Authors:** Lamiaa A. Ahmed, Ahmed F. Mohamed, Enas A. Abd El-Haleim, Dalia M. El-Tanbouly

**Affiliations:** Department of Pharmacology & Toxicology, Cairo University, Cairo, Egypt

**Keywords:** Akt, myocardial fibrosis, platelet activating factor, rupatadine, T helper 17

## Abstract

Disruption of Th17/Tregs homeostasis plays a crucial role in governing the immune response during myocardial fibrosis and its progression to heart failure. The present study aimed to assess for the first time the possible protection afforded by rupatadine against isoproterenol-induced heart failure in rats. It also explored the role of PI3k/Akt as a possible mechanistic pathway, through which rupatadine could modulate Th17/Tregs balance to display its effect. Isoproterenol (85 and 170 mg/kg/day) was injected subcutaneously for 2 successive days, respectively and rupatadine (4 mg/kg/day) was then given orally for 14 days with or without wortmannin (PI3K/Akt inhibitor). Rupatadine succeeded to completely ameliorate isoproterenol-induced cardiac dysfunction as demonstrated by improvements of electrocardiographic and echocardiographic measurements. Moreover, rupatadine prevented the marked elevation of PAF and oxidative stress in addition to Th17 promoting cytokines (IL-6, IL-23, and TGF-β). Accordingly, rupatadine prevented Th17 stimulation or expansion as indicated by increased Foxp3/RORγt ratio and decreased production of its pro-inflammatory cytokine (IL-17). Rupatadine treatment mitigated isoproterenol-induced activation of STAT-3 signaling and the imbalance in *p*-Akt/total Akt ratio affording marked decrease in atrogin-1 and apoptotic biomarkers. Finally, this therapy was effective in averting cardiac troponin loss and reverting the histological alterations as assessed by myocardial fibrosis and hypertrophy grading. Contrariwise, co-administration of wortmannin mostly attenuated the protective effects of rupatadine affording more or less similar results to that of isoproterenol-untreated rats. In conclusion, rupatadine could be an effective therapy against the development of isoproterenol-induced heart failure where PI3K/Akt pathway seems to play a crucial role in its protective effect.

## Introduction

Heart failure (HF) is a common pathophysiologic event following many cardiovascular disease conditions including cardiac surgery as well as myocardial ischemia, myocardial infarction (MI) and hypertrophy (Wang et al., 2017). These events usually begin with myocardial damage and fibrosis which increase the stiffness of heart muscle and cause subsequent mechanical and electrical dysfunction leading to arrhythmia, HF, and even sudden death (Brilla et al., 1991). The rat model of isoproterenol (ISO)-induced myocardial damage is a reliable, reproducible, and non-invasive standardized model which is associated with arrhythmias, myocyte loss, and fibrosis, with progression to HF (Szabo et al., 1975; Krenek et al., 2009). The pathophysiological and morphological aberrations produced in this experimental model are comparable with those that occur in human (Afroz et al., 2016).

Several injury-triggered events play a crucial role in the pathogenesis of HF. Upon cardiac injury, sustained inflammatory response plays a key role in the process of cardiac fibrosis and remodeling with progression to left ventricular hypertrophy and HF (Diwan et al., 2005). Platelet-activating factor (PAF) is a potent lipid mediator of inflammation which affords immunomodulatory effects in the pathogenesis of inflammatory disorders and fibrosis (Kelesidis et al., 2015). PAF acts via a G-protein-coupled receptor (PAFR), upregulating the secretion of a variety of cytokines and promoting neutrophils chemotaxis through platelet-leukocytes conjugation (Sugano et al., 2001).

PAF/PAF-R interaction leads to T helper 17 (Th17) differentiation through creation of a proinflammatory environment that would skew cytokine production in favor of Th17 development over regulatory T-cells (Tregs) (Edwards and Constantinescu, 2009). Due to the broad distributions of interleukin (IL)-17 receptors, they induce a dual massive profibrotic and proinflammatory response (Korn et al., 2009). Former studies have highlighted the detrimental effect of IL-17 during liver (Wang et al., 2011; Tan et al., 2013; Sun et al., 2014) and pulmonary fibrosis (Mi et al., 2011; Zhang et al., 2019). IL-17 was previously claimed to be the major driving force for cardiac remodeling post-myocarditis (Liu et al., 2012). IL-17 also regulates the production of transforming growth factor-beta 1 (TGF-β1), which induces activation of cardiac fibroblast into myofibroblasts, and further facilitates the differentiation of Th17 cells via signal transducer and activator of transcription-3 (STAT3) signaling pathway (Meng et al., 2012). Despite the emphatic role of IL-17-producing cells in the progression of HF, the exact intracellular molecular mechanisms favoring their preferential differentiation, especially in response to PAF, are not utterly characterized.

One of the most vital downstream signaling of T cells receptor is the phosphoinositide 3-kinase/protein kinase B (PI3K/Akt) (Jones et al., 2000; Cantrell, 2002; Kane and Weiss, 2003). This pathway plays an important role in a diversity of physiological and pathogenic processes, including inflammation and cell apoptosis (Wei et al., 2017). The activation of the PI3K/Akt pathway was previously revealed to protect against ISO-induced myocardial injury (Ke et al., 2017). Furthermore, in HF, the decrease in the activity of Akt was associated with induction of atrogin-1 (a marker of muscle atrophy) that favors heart muscle loss and left ventricular dysfunction (Galasso et al., 2010).

Rupatadine (RUP) is a novel second-generation antihistamine which possesses anti-inflammatory effects in addition to dual blockade of histaminic (H1) and PAF-receptors (Mullol et al., 2008). Unlike several antihistamines, preclinical and clinical studies point to its cardiac safety with no proarrhythmic potential upon its use (Donado et al., 2010). In a previous experimental study, RUP promoted the resolution of inflammation and fibrosis in bleomycin-and silica-induced pulmonary fibrosis (Lv et al., 2013). Importantly, interaction between PI3K/Akt signaling and PAF has been clearly shown in inflammatory and fibrotic diseases (Lu et al., 2008) where inhibition of PI3K/Akt signaling was demonstrated to play an important role in preclinical models of cardiac inflammation and HF (Ghigo et al., 2011). Thus, the present study, was conducted to identify PI3k/Akt as a possible mechanistic pathway, through which RUP could modulate Th17/Treg balance to display a protective effect against ISO-induced HF in rats.

## Material and Methods

### Animals

Adult male Wistar albino rats, weighing 170–190 g, were procured from the National Research Center, Cairo, Egypt. Animals were housed at animal facility of Faculty of Pharmacy, Cairo University and allowed free access to standard rat chow diet and water. They were kept at temperature of 25 ± 2°C with a relative humidity of 60 ± 10% and a constant light cycle. This study complied with the Guide for the Care and Use of Laboratory Animals published by the US National Institutes of Health (NIH Publication No. 85-23, revised 2011) and was approved by the Ethics Committee for Animal Experimentation at Faculty of Pharmacy, Cairo University (Permit number: 2770).

### Chemicals

ISO hydrochloride and wortmannin were procured from Sigma–Aldrich Company, United States. RUP was obtained from ATCO Pharma, Egypt. All other chemicals and reagents, unless specified, were obtained from Sigma-Aldrich, United States.

### Induction of Heart Failure in Rats

For induction of HF, rats were injected subcutaneously with ISO for two successive days at different doses (85 and 170 mg/kg, respectively) (Mohamed et al., 2015). Only animals exhibited electrocardiographic deviations (ST elevation) and high serum creatine kinase-MB 24 h after the 2^nd^ ISO injection, were chosen to complete the study.

### Experimental Design

Rats were randomly allocated into four groups, 11 animals each. Group I comprised normal rats that received normal saline. The remaining three groups comprised rats showing signs of myocardial damage and treated starting from the day after ISO injections for 2 weeks daily as follows: group II received normal saline, group III: received RUP (4 mg/kg/day, p. o.) where this dose was selected based on a previous study demonstrating its efficacy as an anti-fibrotic agent in lung fibrosis in rats (Lv et al., 2013) and group IV: received wortmannin (15 μg/kg/day, i. v.) as a PI3K/Akt inhibitor (Sayed et al., 2020) 30 min before RUP administration.

### Hemodynamic Measurements

After 14 days of treatment, electrocardiogram (ECG) was recorded by means of subcutaneous peripheral limb electrodes (HPM 7100, Fukuda Denshi, Tokyo, Japan) inserted under anesthesia using thiopental (50 mg/kg, i. p.). ECG recordings were used to determine heart rate (HR), QT interval and QRS duration. Meanwhile, left ventricular (LV) function was assessed with 12.5- MHz ultrasound probe using Honda HS-2200 V(Tokyo, Japan). Both left ventricular end-diastolic diameter (LVEDD), left ventricular end-systolic diameter (LVESD) and ejection fraction percentage (EF%) were automatically calculated in M-Mode of long-axis parasternal view and provided by the built-in software. Each parameter was assessed as an average over three cardiac cycles (Nagaya et al., 2004).

### Biochemical Measurements

After being subjected to hemodynamic tests, rats were sacrificed by decapitation and blood was withdrawn for serum collection. Hearts of 5 rats form each group were used for histopathological evaluation. Whole ventricles of the remaining rats were rapidly dissected, washed, dried and weighed. A part from each ventricle was homogenized in cold phosphate buffer saline (PBS; pH = 7.4) to prepare 10% homogenate. The other part was kept frozen at −80°C to be used in western blot analysis.

#### Enzyme Linked Immunosorbent Assay

Brain natriuretic peptide (BNP) as a diagnostic and prognostic marker of HF was measured in serum using ELISA kit (MyBioSource, Inc. San Diego, CA, United States). In addition, the following parameters were estimated in tissue homogenates using the corresponding ELISA kits; IL-17 (Sinogeneclon Co., Ltd., China), IL-6 (Chongqing Biospes Co., Ltd., China), P-STAT-3 (Creative Diagnostics; NY, United States), Bax, Bcl2, IL-23, TGF-β, PAF, and atrogin-1 (MyBioSource, Inc. San Diego, CA, United States). A specific ELISA Kit (ab176657, Cambridge, MA, United States) were used to measure both *p*-Akt (Ser473) and total Akt in tissue homogenate.

#### Oxidative Stress Markers

The lipid peroxidation products were assessed colorimetrically by determining the levels of thiobarbituric acid reactive substances (TBARS) in cardiac tissues using a colorimetric assay kit (Biodiagnostic, Egypt). Meanwhile, the antioxidant status of myocardial tissues, including reduced glutathione (GSH), superoxide dismutase (SOD) and catalase, was assessed by using commercially available standard kits (Biodiagnostic, Egypt) according to the manufacturers’ instructions.

#### Determination of Caspase-3 Activity

Caspase-3 activity (the key executioner enzyme in both extrinsic and intrinsic apoptosis) was estimated using caspase-3 colorimetric assay kit (R&D Systems, Inc. United States) where absorbance was read at 405 nm using a microplate reader (BioTek instruments, United States). The results were expressed as fold change in optical density relative to the normal group.

The protein content was determined in all previously mentioned parameters using the method of Lowry et al. (1951).

#### Western Blot Analysis

Part of the ventricle was homogenized in lysis buffer and protein levels were assessed using a Bicinchoninic acid (BCA) protein assay kit (Thermo Fisher Scientific Inc. United States). Protein expression was estimated as previously described (Ahmed et al., 2014) using troponin I primary antibody (Thermofisher scientific, United States, Cat.# MA1-20112), troponin T primary antibody (Thermofisher scientific, United States, Cat.# MA1-24611), forkhead/winged helix transcription factor P3 (FoxP3) primary antibody (Thermofisher scientific, United States, Cat.# PA5-23169), retinoic-acid-related orphan receptor (ROR)-γt primary antibody (Thermofisher scientific, United States, Cat.# PA5-23148) and horseradish peroxidase (HRP)-conjugated goat anti-rabbit secondary antibody (Thermofisher scientific, United States, Cat.# 31460). The amount of protein was quantified by densitometric analysis of the autoradiograms using a scanning laser densitometer (Biomed Instrument Inc. United States). Results were normalized to *β*-actin and expressed as fold change to the normal group.

### Histopathological Analysis

Cardiac tissues were fixed in 10% formalin and embedded in paraffin wax. In order to assess the degree myocardial fibrosis, tissue sections (5 µm) were stained with Masson trichrome to identify collagen fiber in cardiac tissue using an image analyzer (Leica Qwin 550, Germany). The percentage of fibrosis for each group was calculated as the average of randomly chosen 10 fields from each section (Berry et al., 2006). Sections of 5 µm were also cut and stained with hematoxylin and eosin (H&E) to assess hypertrophy and myocardial damage. Myocardial damage was evaluated as demonstrated by Zhang et al. (2008) using a semi-quantitative grading scale of 0–5. All histological changes were evaluated by a pathologist unaware of different groups examined.

### Statistical Analysis

All results are reported as means ± SD. GraphPad Prism software (version 6.04) was used to perform all statistical analyses. The analysis of all data was done using One-way ANOVA then Tukey multiple comparisons as a post hoc test except histological score of damage which was done using non-parametric One-Way ANOVA followed by Dunn’s multiple comparison test. *p* value <0.05 was considered as a significant difference.

## Results

### Heart Weight Index (HWI) and Hemodynamic Measurements

ISO-induced HF caused a significant increase in HWI indicating myocardial hypertrophy. Treatment with RUP completely reverted changes in HWI, an effect that was abolished by co-administration of wortmannin, a selective inhibitor of PI3K/Akt pathway (Table 1). Furthermore, ISO administration induced conduction and contraction abnormalities as indicated by significant increase in QT interval, QRS duration, LVEDD, and LVESD measurements together with a significant decrease in HR and EF%. These results were associated with a marked rise in serum level of BNP confirming the presence of cardiac dysfunction and HF. Conversely, RUP succeeded to improve eletrocardiographic and echocardiographic perturbations in addition to BNP level. These results were mostly reverted by addition of PI3K/Akt inhibitor (Table1).

**TABLE 1:** Effect of RUP with or without wortmannin on HWI electrocardiographic and echocardiographic parameters as well as serum BNP level in ISO-induced HF in rats.

	Normal	ISO	ISO/Rup	ISO/Rup/Wor
HWI (mg/g)	2.74 ± 0.07	3.71 ± 0.14^a^	2.74 ± 0.11^b^	3.68 ± 0.11^a,c^
HR (bpm)	453.8 ± 15.5	349.8 ± 9.5^a^	451 ± 8.2^b^	372.7 ± 29^a,c^
QT (ms)	81.3 ± 5.3	127.3 ± 10.1^a^	76 ± 5.2^b^	113.3 ± 11.2^c^
QRS (ms)	12.6 ± 1.9	25.6 ± 1.5^a^	10 ± 0.7^b^	12.7 ± 1.5^b^
LVESD (mm)	5.3 ± 0.05	8.9 ± 0.07^a^	6.5 ± 0.06^a,b^	8.5 ± 0.05^a,b,c^
LVEDD (mm)	7.8 ± 0.08	10.1 ± 0.06^a^	8.2 ± 0.09^a,b^	9.8 ± 0.04^a,b,c^
EF (%)	65.0 ± 1.0	31.6 ± 1.2^a^	60.3 ± 0.6^a,b^	52.7 ± 1.2^a,b,c^
BNP	115.5 ± 1.88	301.7 ± 24.8^a^	188 ± 3.56^a,b^	272.8 ± 20.77^a,c^

Each value represents the mean of six experiments ±SD. Statistical analysis was done using One way ANOVA followed by Tukey’s post-hoc test.

^a^ *p* <0.05 vs. normal.

^b^ p <0.05 vs. ISO.

^c^ *p* <0.05 vs. RUP.

BNP, brain natriuretic peptide; EF, ejection fraction; HR, heart rate; HW, heart weight; HWI, heart weight index; ISO, isoproterenol; LVESD, left ventricular end systolic diameter; LVEDD, left ventricular end diastolic diameter; Rup, rupatadine; Wor, wortmannin.

### Platelet Activating Factor, Oxidative Stress and Th17 Promoting Cytokines (IL-6, IL-23 and TGF-β)

**FIGURE 1:**
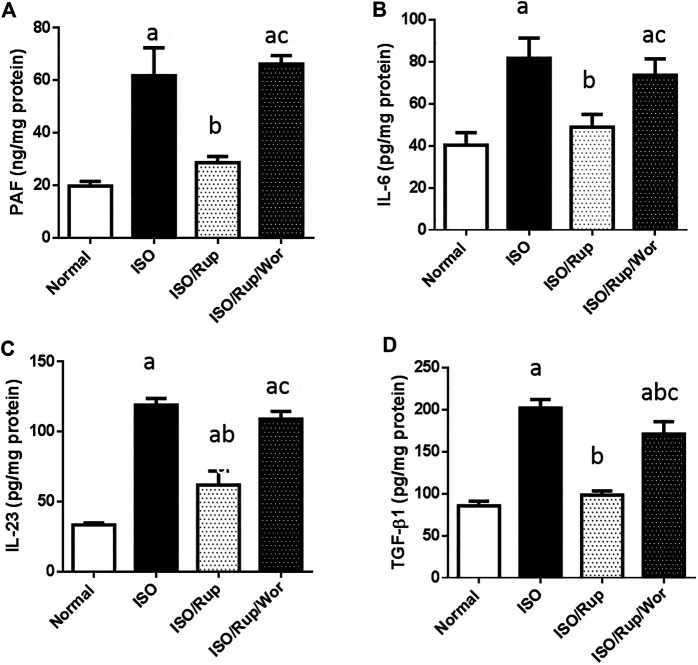
Effect of RUP with or without wortmannin on ISO-induced changes in myocardial contents of **(A)** PAF **(B)** IL-6 **(C)** IL-23, and **(D)** TGF-β. Each value represents the mean of six experiments and error bars represent SD. Statistical analysis was done using One way ANOVA followed by Tukey’s post-hoc test where a *p* < 0.05 vs. normal, b *p* < 0.05 vs. ISO, c *p* < 0.05 vs. RUP.

**FIGURE 2:**
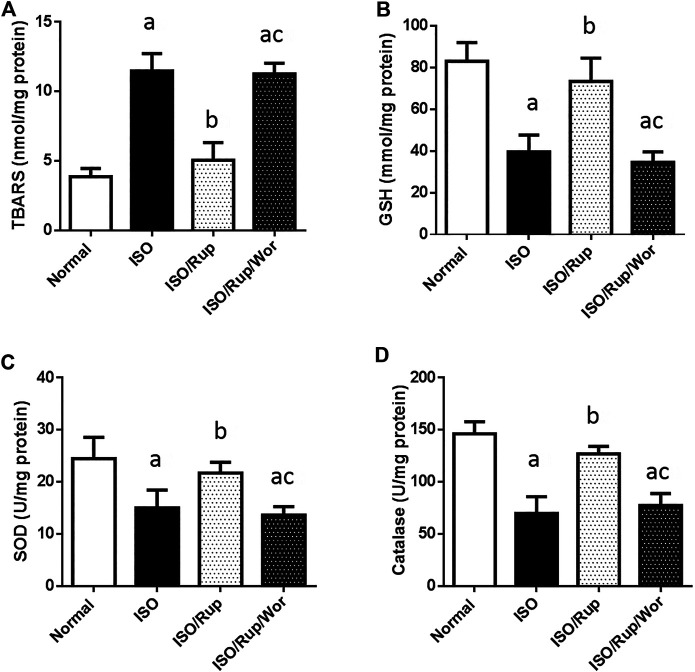
Effect of RUP with or without wortmannin on ISO-induced changes in myocardial contents of **(A)** TBARS **(B)** GSH **(C)** SOD and **(D)** catalase. Each value represents the mean of six experiments and error bars represent SD. Statistical analysis was done using One way ANOVA followed by Tukey’s post-hoc test where a *p* < 0.05 vs. normal, b *p* < 0.05 vs. ISO, c *p* < 0.05 vs. RUP.

ISO-treated rats showed 3-fold increase in PAF together with significant reduction of antioxidant capacity of cardiac tissues (GSH, SOD and catalase) and significant elevation of the levels of TBARS, IL-6, IL-23, and TGF-β, indicating the activation of oxidative stress, inflammatory and fibrotic pathways. Meanwhile, almost these markers were normalized using RUP treatment. Administration of RUP and wortmannin together significantly reversed the effect of RUP on TGF-β besides complete abolishment of the effect of RUP on oxidative stress markers in addition to IL-6 and IL-23 showing similar results to ISO group (Figures 1, 2).

### Foxp3/RoR-γt Ratio and IL17

**FIGURE 3:**
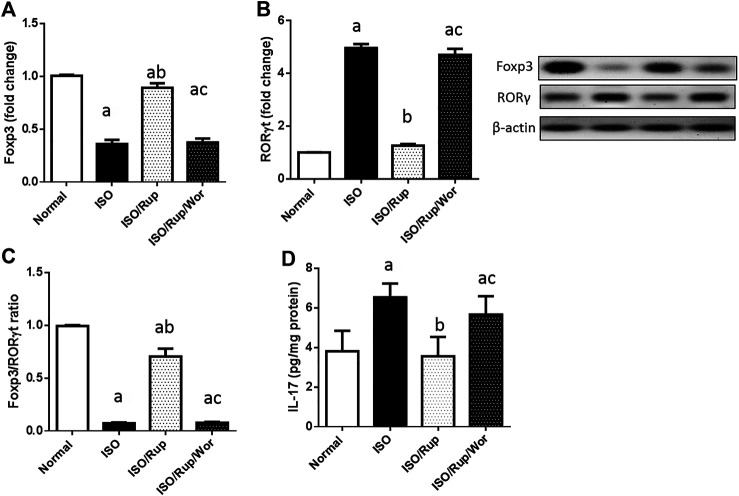
Effect of RUP with or without wortmannin on ISO-induced changes in protein expression of **(A)** Foxp3 and **(B)** RORγt in addition to **(C)** Foxp3/RORγt ratio and myocardial content of **(D)** IL-17. Each value represents the mean of six experiments and error bars represent SD. Statistical analysis was done using One way ANOVA followed by Tukey’s post-hoc test where a *p* < 0.05 vs. normal, b *p* < 0.05 vs. ISO, c *p* < 0.05 vs. RUP.

The elevation of Th17 promoting cytokines was accompanied by a marked reduction in Foxp3/RORγt ratio in ISO-treated rats indicating the expansion of Th17 over Tregs. This was associated with significant increase in the production of its pro-inflammatory cytokine IL-17. Again, administration of RUP succeeded to significantly increase Foxp3/RORγt ratio together with normalization of IL-17 level. On the other hand, there was no significant difference between the results of ISO-treated group and the group received both RUP and wortmannin (Figure 3).

### p-STAT 3 and pAkt/Total Akt Ratio

**FIGURE 4:**
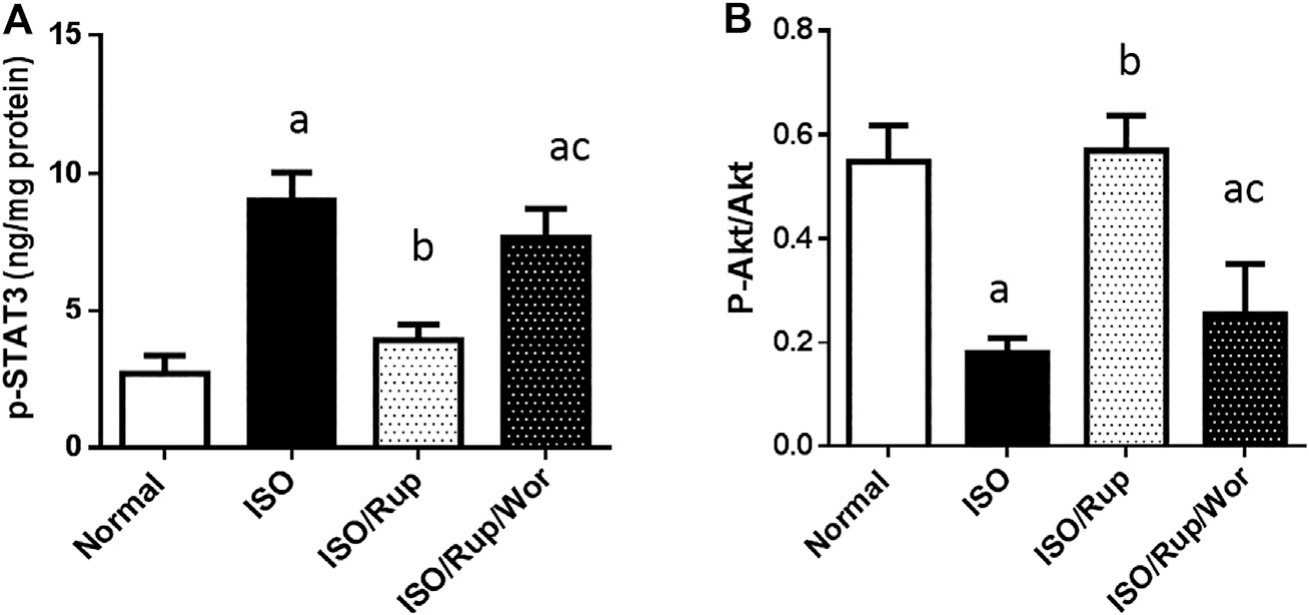
Effect of RUP with or without wortmannin on ISO-induced changes in myocardial contents of **(A)** p-STAT 3 and **(B)** pAkt/total Akt ratio. Each value represents the mean of six experiments and error bars represent SD. Statistical analysis was done using One way ANOVA followed by Tukey’s post-hoc test where a *p* < 0.05 vs. normal, b *p* < 0.05 vs. ISO, c *p* < 0.05 vs. RUP.

Administration of ISO caused the activation of STAT3 signaling as demonstrated by significant rise in the level of p-STAT3. This was correlated with a significant decrease in *p*-Akt/total Akt ratio. RUP treatment prevented these changes whereas these effects were completely attenuated by co-administration of an inhibitor of PI3K/Akt pathway (Figure 4).

### Cardiac Atrogin 1 and Troponin I and T

**FIGURE 5:**
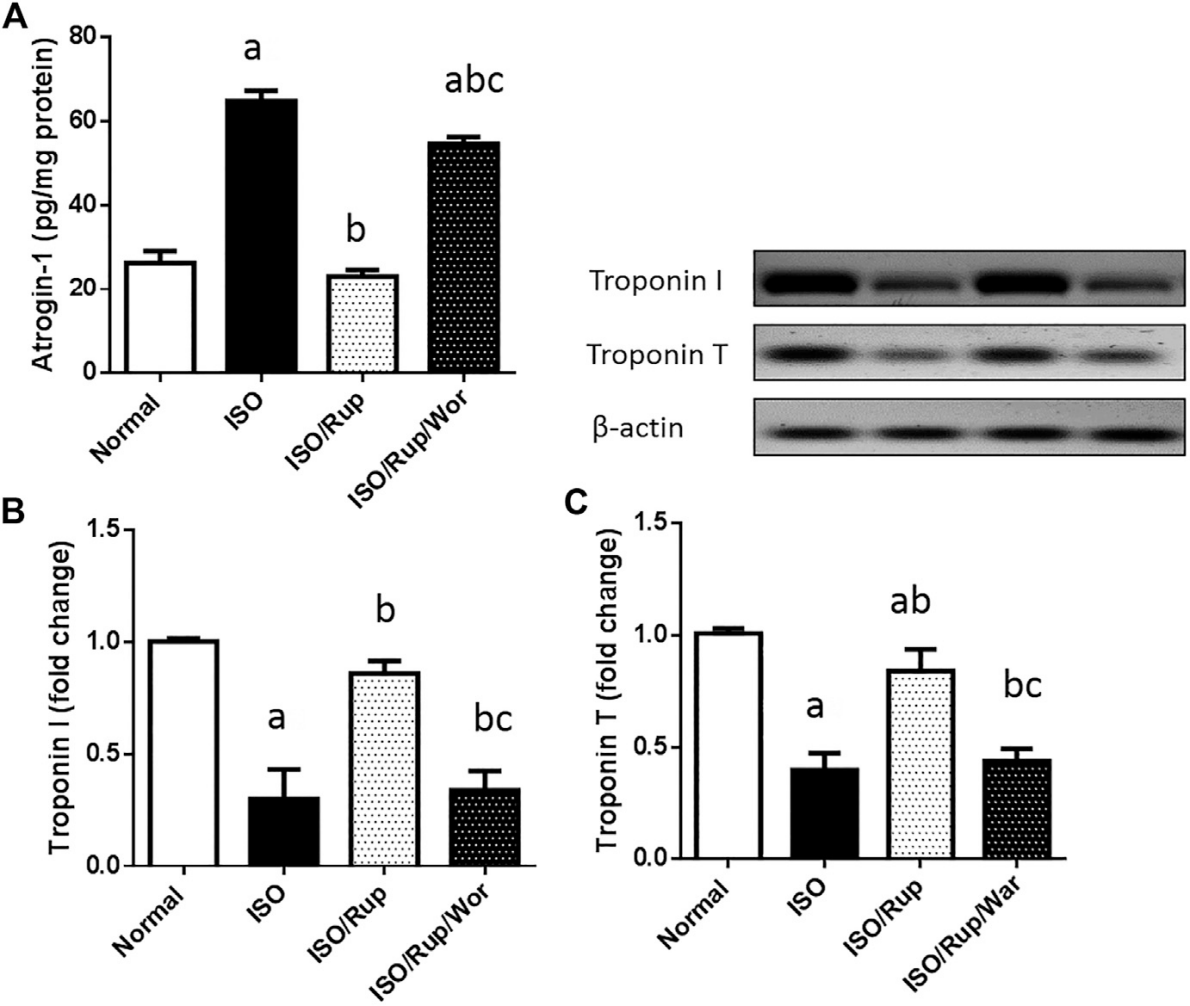
Effect of RUP with or without wortmannin on ISO-induced changes in myocardial content of **(A)** atrogin-1 as well as the protein expression of **(B)** troponin I and **(C)** troponin T. Each value represents the mean of six experiments and error bars represent SD. Statistical analysis was done using One way ANOVA followed by Tukey’s post-hoc test where a *p* <0.05 vs. normal, b *p* <0.05 vs. ISO, c *p* <0.05 vs. RUP.

Compared to normal group, ISO-treated animals showed a marked rise in the level of atrogin-1 with diminution in the protein expression of both troponin I and T. Notably, administration of RUP normalized atrogin 1 content and remarkably increased the protein expression of troponin I and T, where these markers were worsened again upon co-administration of wortmannin (Figure 5).

### Apoptotic Biomarkers

**FIGURE 6:**
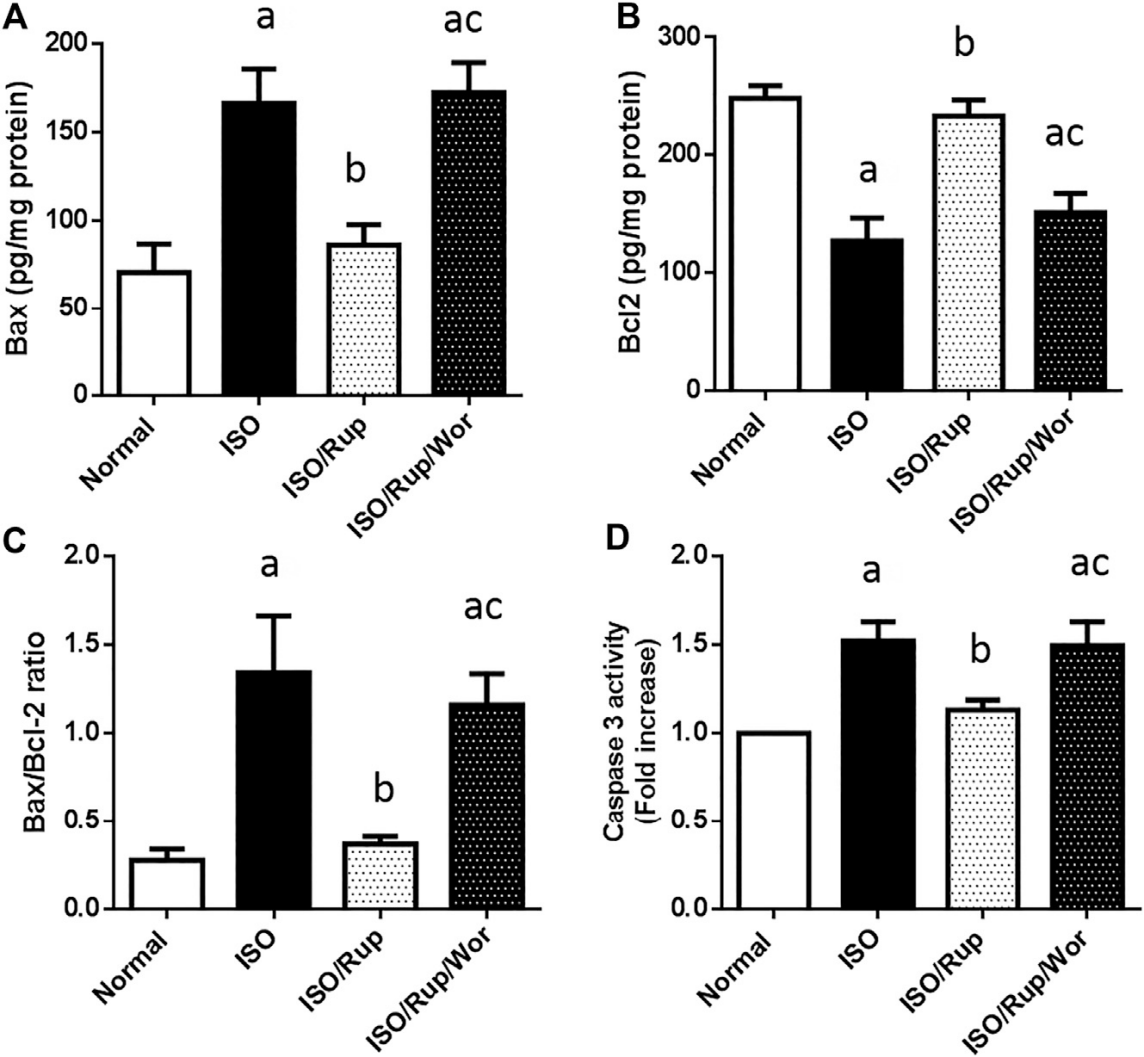
Effect of RUP with or without wortmannin on ISO-induced changes in myocardial contents of **(A)** Bax and **(B)** Bcl2 in addition to **(C)** Bax/Bcl2 ratio and **(D)** caspase-3 activity. Each value represents the mean of six experiments and error bars represent SD. Statistical analysis was done using One way ANOVA followed by Tukey’s post-hoc test where a *p* < 0.05 vs. normal, b *p* < 0.05 vs. ISO, c *p* < 0.05 vs. RUP.

ISO enhanced myocardial apoptotic death as indicated by significant increase in caspase-3 activity and Bax/Bcl2 ratio. Rats treated with RUF exhibited profound decrease in these markers, an effect that was completely abolished by co-administration of wortmannin (Figure 6).

### Histological Examinations of Myocardial Fibrosis and Damage

**FIGURE 7:**
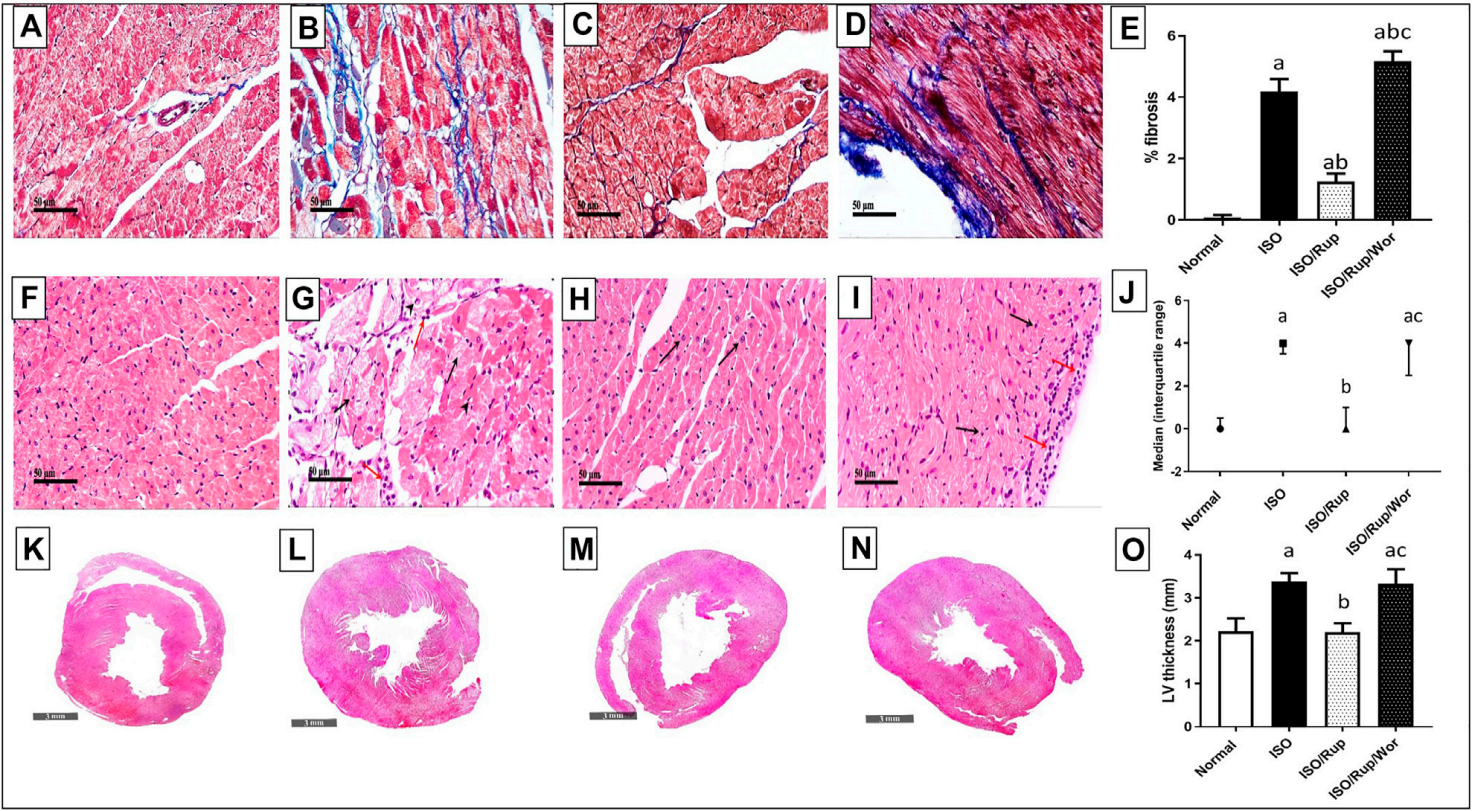
Effect of RUP with or without wortmannin on ISO-induced histological changes. Normal group **(A,F,K)**, ISO group **(B,G,L)**, RUP group **(C,H,M)** and RUP + Wort group **(D,I,N)**. **(A–D)** Specimens stained with Masson’s trichrome for estimation of myocardial fibrosis (blue color). Specimens stained with hematoxylin and eosin for estimation of degree of myocardial damage **(F–I)** and hypertrophy **(K–N)**. Percentage of fibrosis **(E)** and left ventricular (LV) thickness **(O)**. Each value represents the mean of 5 experiments and error bars represent SD. Statistical analysis was done using One way ANOVA followed by Tukey’s post-hoc test. Histopathological scores **(J)** are expressed as the median of 5 animals. Statistical analysis was done using Kruskal-Wallis test followed by Dunn’s test where a *p* < 0.05 vs. normal, b *p* < 0.05 vs. ISO, c *p* < 0.05 vs. RUP.

ISO-treated group showed a significant increase in hypertrophy and the percentage of fibrosis compared to normal group. Similarly, ISO administration caused a marked cardiac muscle damage with a cardiac lesion score of 3. Treatment with RUP significantly attenuated the development of hypertrophy and fibrosis compared to ISO group together with improvement of myocardial architecture giving a cardiac lesion score of 1. Co-administration of wortmannin reversed the protective effects of RUP giving pictures similar to ISO group with a cardiac score of 4 (Figure 7).

## Discussion

The present study emphasizes the efficacy of RUP in interfering with the processes of myocardial fibrosis, cardiac remodeling and ultimately HF. RUP noticeably prevented myocardial damage and hypertrophy where these effects were reflected largely in its ability to reserve cardiac function following ISO injection as shown by electrocardiographic as well as echocardiographic measurements.

Disruption of Th17/Tregs homeostasis plays a crucial role in governing the immune response during HF (Myers et al., 2016). Th17 phenotypes expressing ROR-γt have pivotal roles in tissue inflammation and autoimmunity by releasing IL-17 (Crome et al., 2010; Hammerich et al., 2011; Miossec and Kolls, 2012), while Tregs phenotypes expressing FoxP3 exert an anti-inflammatory function and preserve tolerance to self-antigens (Lehtimäki and Lahesmaa, 2013). Antigen-presenting cells (APCs), such as monocytes secrete the Th17-promoting cytokines; TGF-β1, IL-6, and IL-23 (Evans et al., 2007; Volpe et al., 2008). These cytokines synergize to induce ROR-γt expression, thus supporting Th17 differentiation (Aggarwal et al., 2003; Kolls and Lindén, 2004; Wilson et al., 2007; Manel et al., 2008; Hwang, 2010). Interestingly, co-culture of CD4^+^ T cells with PAF-stimulated APCs, previously developed a Th17 phenotype via endorsing the rapid expression of Th17-promoting cytokines (Drolet et al., 2011). In parallel, during immunoinflammatory reactions, PAF triggers macrophage to liberate reactive oxygen species evoking a state of oxidative burst (Hartung et al., 1983) which contributes to Th17/Tregs imbalance through inducing the pro-inflammatory Th17 cells expansion and inhibiting the anti-inflammatory Treg cells differentiation (Yang et al., 2016). Beside its indirect effect on Th17 via its receptors on APCs, PAF was claimed to have receptors on T cells which are upregulated upon stimulation and may directly drive them toward Th17 phenotype (Calabresse et al., 1992; Edwards and Constantinescu, 2009). Accordingly, PAF affects the response of Th17 cells and mediates its production of IL-17 (Drolet et al., 2011). IL-17 promotes fibrosis by exacerbating the upstream oxidative (Swardfager et al., 2014) and inflammatory responses as well as regulating the downstream activation of fibroblasts (Fang et al., 2016). It increases the secretion of pro-inflammatory cytokines, including IL-1, IL-6, and tumor necrosis factor-alpha (TNF-α), by inflammatory cells which further reinforce the inflammatory response (Meng et al., 2012). Concomitantly, it could provoke the production of TGF-β1, the major fibrogenic factor, which induces the activation of cardiac fibroblast into myofibroblasts (Faust et al., 2009; Mi et al., 2011), and further facilitates the differentiation of Th-17 (Manel et al., 2008). TGF-β also directly stimulates its receptors constitutively expressed on cardiac fibroblasts to promote collagen types I and III production through enhancing phosphorylation and thus nuclear translocation of the transcription factor, STAT3 (Meng et al., 2012). In addition, IL-17 regulates matrix metalloproteinases/tissue inhibitors of matrix metalloproteinases (MMP/TIMP) system which was previously revealed to mediate tissue remodeling in rats with ISO-induced HF as well as in primary human cardiac fibroblasts (Cortez et al., 2007; Feng et al., 2009). Accordingly, IL-17 contributes largely to myocardial interstitial fibrosis as demonstrated herein and previously (Liu et al., 2012; Myers et al., 2016).

In the present study, ISO injection induced a state of Th17/Tregs imbalance with a massive disruption of ventricular PAF, TBARS, GSH, SOD, catalase, TGF-β1, IL-6, IL-23, and IL-17 contents. RUP was effective in increasing Foxp3/RORγt ratio, an effect that was associated with a marked reduction in PAF, oxidative stress and the evoked cytokines. As a result of its effects on both TGF-β and IL-17, the accumulation of collagen and cardiac remodeling were largely mitigated. In harmony with these results, RUP exerted obvious anti-fibrotic effects in rat models of diabetic nephropathy (Hafez et al., 2020) and bleomycin-induced pulmonary fibrosis (Lv et al., 2013).

The influence of RUP on Th17 differentiation could be partially attributed to its ability to block PAF receptors and consequently TGF-β/IL-6/IL-23/IL-17 axis. Coherent with these results, Singh et al. (2011) demonstrated that pretreatment of monocyte-derived Langerhans cells with a PAFR antagonist prevented PAF-prompted RORγt expression by T cells. PAFR blockage decreased also the level of Th17–related cytokines, IL-17, IL-23, and IL-6 in the skin of transgenic mice in addition to reduction of PAF level probably by interfering with its autocrine loop.

It has been reported that enhanced Akt signal could impede the effect of Th17-promoting cytokines on CD4^+^ T cells, instead, it could support Foxp3 expression (Pierau et al., 2009). On the other hand, PAF could inhibit PI3k/Akt signaling pathway via promoting rapid dephosphorylation of various intermediates (Lu et al., 2008). Furthermore, pretreatment of PAF-primed Langerhans cells with STAT3 inhibitors totally prevented RORγt production in CD4^+^ T cells (Drolet et al., 2011). In the same context, STAT3 directly binds to IL-17 promoters after being translocated to the nucleus in response to PAF stimulation (Chen et al., 2006). STAT3 phosphorylation could be dramatically inhibited, however, by a PAF blocker (Deo et al., 2002, 2004). These observations suggest the possible interplay between Akt and STAT3 in regulating PAF-induced Th17 activity outbreak. Consistent with these observations, the increase in PAF following ISO injection, in the current study, was associated with a marked reduction in *p*-Akt along with an obvious upsurge in p-STAT3. On the other hand, RUP succeeded in reversing these actions to accomplish its inhibitory effect on Th17 phenotypes.

Beside its negative effect on Akt phosphorylation, wortmannin, a PI3k inhibitor, interfered with the effect of RUP on P-STAT3 in the current work. A possible explanation of this effect is the ability of Akt to inhibit glycogen synthase kinase 3-β (Beurel, 2014) whose activity is positively correlated with Th-17 differentiation via promoting STAT3 phosphorylation (Beurel et al., 2011). Consequently, wortmannin impeded RUP effects on IL-17 and RORγt expression as well as its inhibitory actions on IL-6 and, IL-23, TGF-β, PAF, and oxidative stress. The spike in PAF observed following pretreatment with wortmannin could be probably explained by reducing its catabolism by PAF-acetylhydrolase secreted mainly by monocytes (Stafforini, 2009). Inhibition of Akt pathway reduces nuclear translocation of specificity protein 1 activation in monocytes (Lee et al., 2015), the main transcription factor for this catabolizing enzyme (Stafforini, 2009). The current results affirm that the inhibitory effect of RUP on Th17- promoting cytokines was established mainly via its complementary boosting effect on Akt pathway.

Impaired PI3K/Akt signaling was previously accused of being the chief mechanism mediating the pro-apoptotic effect of PAF (Lu et al., 2008). Interestingly, IL-17 could act directly on its receptors expressed on cardiomyocyte to induce apoptosis via increasing the Bax/Bcl-2 ratio (Liao et al., 2012). In addition, protein-ubiquitination by the overexpressed atrogin-1 in response to impaired Akt activity contributes mostly to myocardial remodeling and degeneration (Galasso et al., 2010). In the present study, the impaired Akt phosphorylation was accompanied by an obvious increase in caspase-3 activity, Bax/Bcl2 ratio and atrogin-1 levels along with a significant loss of cardiac troponin I and T in ISO group. Coherent with these results, Li et al. (2018) reported that sustained activation of Akt signaling pathway could inhibit cardiomyocyte apoptosis following ISO-induced HF. Additionally, reduction of Akt phosphorylation reported in patients with HF was positively correlated with heart muscle degeneration and left ventricular dysfunction (Galasso et al., 2010). Conversely, rats treated with RUP showed normal atrogin-1 level, caspase-3 activity and Bax/Bcl2 ratio with a marked increase in troponin I and T contents. These effects were mostly abolished by wortmannin advocating that Akt pathway mediates the protective effect of RUP on cardiomyocyte against apoptosis and degeneration. Activated Akt could diminish the pro-apoptotic (Bad and Bax) factors, while enhancing the anti-apoptotic (Bcl-2) factor (Atif et al., 2015). It also hampers the release of p53, which incites apoptosis under different conditions of cellular stress (Ji et al., 2012). In addition, Akt interferes with cytochrome c–induced caspase-9 and -3 activation at the post-mitochondrial level to pin down its antiapoptotic effect (Zhou et al., 2000).

Importantly, though histamine could induce pulmonary fibroblast proliferation via H1 receptor (Veerappan et al., 2013), H2 receptors are widely expressed on cardiac myocytes, endothelial cells as well as fibroblasts (Leary et al., 2018; Zhang et al., 2018). Ample evidences demonstrate that H2 receptors blockers could hinder HF progression by reducing myocardial fibrosis and apoptosis (Kim et al., 2006; Takahama et al., 2010; Zeng et al., 2014; Zhang et al., 2018). In addition, histamine binding to H4 receptor on Th17 cells promotes their overall stimulation (Mommert et al., 2012). Based on the aforementioned results, the beneficial effects of RUP observed herein could be attributed merely to its PAF blockade activity.

In conclusion, the present study offers profound insights into the role of RUP in mitigating ISO-induced HF. These effects could be related mainly to the anti-fibrotic properties of RUP through interruption of PAF-induced oxidative stress and TGF-β/IL-6/IL-23/IL-17 axis via its complementary boosting effect on Akt pathway.

## Data Availability

The raw data supporting the conclusions of this article will be made available by the authors, without undue reservation, to any qualified researcher.

## Abbreviations

APCs, antigen-presenting cells; BCA, Bicinchoninic acid; BNP, Brain natriuretic peptide; ECG, electrocardiogram; EF%, ejection fraction percentage; FoxP3, forkhead/winged helix transcription factor P3; H&E, hematoxylin and eosin; HF, heart failure; HR, heart rate; H receptor, histaminic receptor; HWI, heart weight index; IL, interleukin; ISO, isoproterenol; LVEDD, left ventricular end-diastolic diameter; LVESD, left ventricular end-systolic diameter; MI, myocardial infarction; MMP/TIMP, matrix metalloproteinases/tissue inhibitors of matrix metalloproteinases; PAF, platelet activating factor; PBS, phosphate buffer saline; PI3K/Akt, phosphoinositide 3-kinase/ protein kinase B; ROR-γt, retinoic-acid-related orphan receptor-γt; RUP, Rupatadine; Th17, T helper 17; TGF-β, transforming growth factor-beta; TNF-α, tumor necrosis factor-alpha; Tregs, regulatory T-cells; STAT3, signal transducer and activator of transcription 3.
