# Supplementary figures and images for: Boosting Akt Pathway by Rupatadine Modulates Th17/Tregs Balance for Attenuation of Isoproterenol-Induced Heart Failure in Rats
Source: Front Pharmacol. 2021 Apr 30;12:651150. doi: 10.3389/fphar.2021.651150 (PMC8121023; doi:10.3389/fphar.2021.651150)

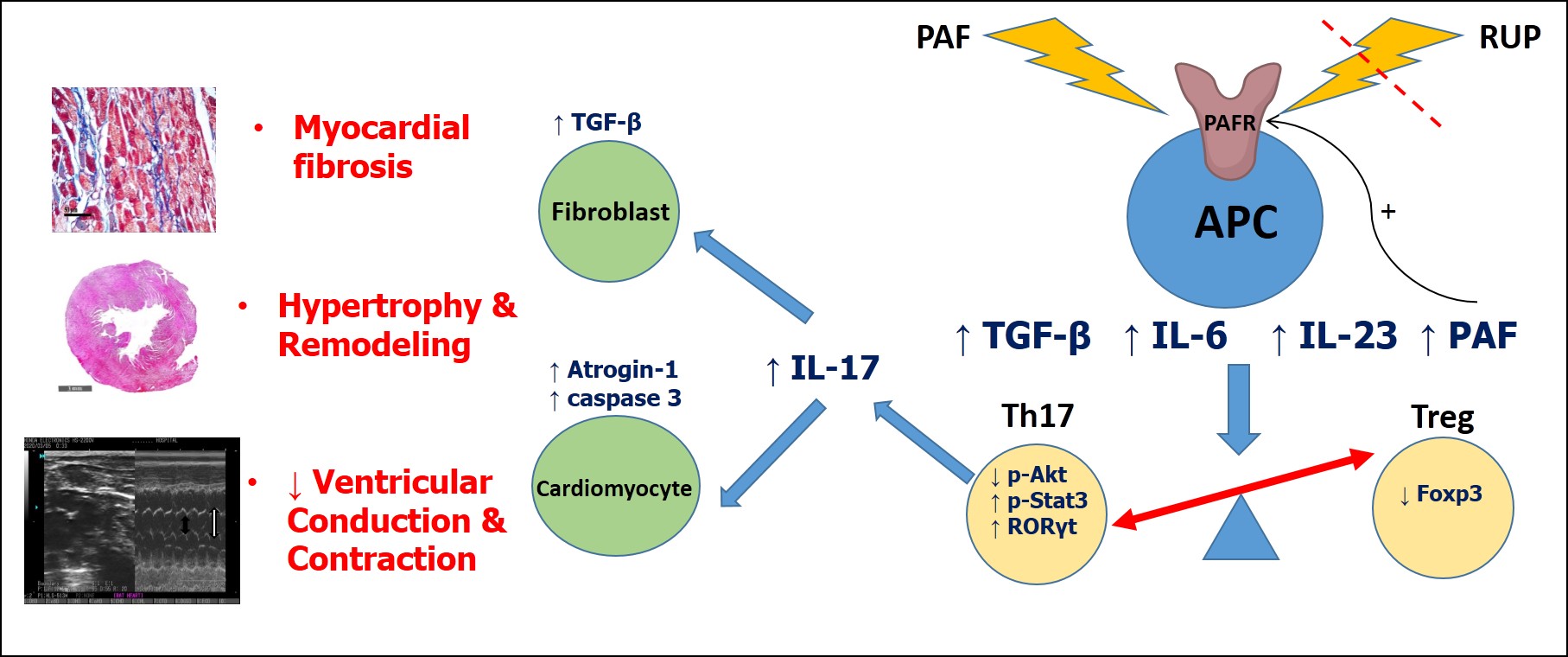

Supplement: Supplementary file 1 [file Image1.JPEG]
